# Identification of factors impairing exercise capacity after severe COVID-19 pulmonary infection: a 3-month follow-up of prospective COVulnerability cohort

**DOI:** 10.1186/s12931-022-01977-z

**Published:** 2022-03-22

**Authors:** Bruno Ribeiro Baptista, Thomas d’Humières, Frédéric Schlemmer, Inès Bendib, Grégoire Justeau, Lara Al-Assaad, Mouna Hachem, Rebecca Codiat, Benjamin Bardel, Laure Abou Chakra, Thibaut Belmondo, Etienne Audureau, Sophie Hue, Armand Mekontso-Dessap, Geneviève Derumeaux, Laurent Boyer

**Affiliations:** 1 Université Paris Est Créteil (UPEC), FHU SENEC, IMRB, INSERM, Créteil, France; 2 Département de Physiologie-Explorations Fonctionnelles, Hôpitaux Universitaires Henri Mondor, AP-HP, Avenue Maréchal de Lattre de Tassigny, Créteil, France; 3 Unité de Pneumologie, Service de Médecine Intensive Réanimation, Hôpitaux Universitaires Henri Mondor, Assistance Publique-Hôpitaux de Paris, Créteil, France; 4 Médecine Intensive Réanimation, Hôpitaux Universitaires Henri Mondor, AP-HP, Créteil, France; 5 Département d’Hématologie et d’Immunologie Biologiques, AP-HP, Hôpitaux Universitaires Henri Mondor, Créteil, France; 6 Biostatistics Department, Henri Mondor Hospital, Assistance Publique Hôpitaux de Paris, Créteil, France; 7 CEpiA IMRB U955, FHU SENEC, Université Paris Est (UPEC), Créteil, France

**Keywords:** SARS-CoV-2, COVID-19, Cardiopulmonary exercise testing, Pulmonary function, Skeletal muscle, Sarcopenia

## Abstract

**Background:**

Patient hospitalized for coronavirus disease 2019 (COVID-19) pulmonary infection can have sequelae such as impaired exercise capacity. We aimed to determine the frequency of long-term exercise capacity limitation in survivors of severe COVID-19 pulmonary infection and the factors associated with this limitation.

**Methods:**

Patients with severe COVID-19 pulmonary infection were enrolled 3 months after hospital discharge in COVulnerability, a prospective cohort. They underwent cardiopulmonary exercise testing, pulmonary function test, echocardiography, and skeletal muscle mass evaluation.

**Results:**

Among 105 patients included, 35% had a reduced exercise capacity (VO_2_peak < 80% of predicted). Compared to patients with a normal exercise capacity, patients with reduced exercise capacity were more often men (89.2% vs. 67.6%, p = 0.015), with diabetes (45.9% vs. 17.6%, p = 0.002) and renal dysfunction (21.6% vs. 17.6%, p = 0.006), but did not differ in terms of initial acute disease severity. An altered exercise capacity was associated with an impaired respiratory function as assessed by a decrease in forced vital capacity (p < 0.0001), FEV1 (p < 0.0001), total lung capacity (p < 0.0001) and DL_CO_ (p = 0.015). Moreover, we uncovered a decrease of muscular mass index and grip test in the reduced exercise capacity group (p = 0.001 and p = 0.047 respectively), whilst 38.9% of patients with low exercise capacity had a sarcopenia, compared to 10.9% in those with normal exercise capacity (p = 0.001). Myocardial function was normal with similar systolic and diastolic parameters between groups whilst reduced exercise capacity was associated with a slightly shorter pulmonary acceleration time, despite no pulmonary hypertension.

**Conclusion:**

Three months after a severe COVID-19 pulmonary infection, more than one third of patients had an impairment of exercise capacity which was associated with a reduced pulmonary function, a reduced skeletal muscle mass and function but without any significant impairment in cardiac function.

**Supplementary Information:**

The online version contains supplementary material available at 10.1186/s12931-022-01977-z.

## Background

The pandemic of coronavirus disease 2019 (COVID-19) caused by the severe acute respiratory syndrome coronavirus 2 (SARS-CoV-2) represents the greatest global public health crisis of the last decades. Most of the knowledge about COVID-19, including its clinical manifestations and early evolution comes from studies focusing on the acute infection phase [1, 2] or short-term convalescence phase [3]. Whilst pulmonary sequelae have been extensively analyzed, few studies focused on factors contributing to impaired exercise capacity.

Indeed, pulmonary alteration such as reduction of diffusing capacity and restrictive pattern have been reported in up to one third of COVID-19 survivors, as previously described in other coronavirus pneumonia such as severe acute respiratory syndrome (SARS) or middle east respiratory syndrome (MERS) [4–9]. Recently, exercise capacity limitation has also emerged as a frequent complication leading to long-term disability in the context of COVID-19 infection [10, 11]. The diagnosis of altered exercise capacity and the understanding of the underlying factors are crucial for patients since they may benefit early from a personalized rehabilitation program. Clinical symptoms and 6-min walk test (6-MWT) may fail to detect such a reduced exercise capacity, whilst cardiopulmonary exercise test (CPET) may unmask exercise limitation.

To decipher the mechanisms underlying the exercise capacity limitation, we conducted a comprehensive prospective evaluation in severe COVID-19 pulmonary infection survivors 3 months after hospital discharge in COVulnerability cohort. More specifically, we performed in addition to CPET and 6-MWT, lung function test, echocardiography, skeletal muscle mass and function evaluation, and measured circulatory inflammatory biomarkers.

## Methods

### Study design and participant

COVulnerability is an ongoing prospective monocentric study, conducted at Henri Mondor Hospital, APHP, Creteil, France. Patient were included between March 2020 and July 2021 when diagnosed with severe COVID-19 pulmonary infection [12], confirmed by positive polymerase chain reaction or serology, hospitalized at intensive care unit (ICU) or at conventional care unit for more than 7 days and with oxygen therapy during hospitalization (> 3 l/min). As part of routine clinical care, a follow-up visit was scheduled 3 months after hospital discharge for a comprehensive evaluation. Patients were excluded if they were under 18 years old or pregnant. The study was approved as part of routine clinical care by ethical committee *Comité consultatif sur le traitement de l'information en matière de recherche* (*C.C.T.I.R.S.*) of the Henri Mondor Hospital. An agreement was obtained from patients prior to CPET, pulmonary function tests and echocardiography at rest.

Demographics, clinical and laboratory findings upon hospital admission, during hospital stay and 3 months post hospital discharge were collected including medical history before COVID-19 infection, smoking status (pack-years), body mass index (BMI) and severity of the acute COVID-19 infection (use of high-flow nasal cannula (HFNC), invasive mechanical ventilation, extracorporeal membrane oxygenation (ECMO)). Assessment of COVID-19-related long-term organ damage was evaluated and included lung function at rest, exercise testing with VO_2_peak measurement, transthoracic echocardiography at rest. Skeletal muscle testing (grip and pinch test) was assessed using a standard handgrip dynamometer and pinch gauge (Baseline Evaluation Instrument, NY, USA). Appendicular skeletal muscle mass (ASMM) was determined using dual-energy X-ray absorptiometry (Lunar iDXA, GE Healthcare, UK) as the fat-free soft-tissue masses of the arms and legs divided by height squared [13] and ASMM index (ASMMI) was then computed as ASMM divided by height squared. The cutoff for defining sarcopenia was two standard deviations below the mean sex-specific ASMMI values in the Rosetta Study (5.45 for females and 7.26 for males), as proposed by Baumgartner et al. [13]. Biological analysis included kidney evaluation by creatinine concentrations and inflammatory evaluation (C reactive protein (CRP), IL6, IL8, TNF).

### Cardiopulmonary test exercise protocol

The instructions given to the subjects were the routine instructions sent to patients coming to the laboratory for clinical exercise testing [14]. Symptom-limited CPET was performed using an electronically braked cycle ergometer (Jaeger Vyntus CPX), under the supervision of a physician with defined criteria for stopping, such as serious cardiac arrhythmias, hypotension, electrocardiographic changes (ST-segment and T-wave changes, arrhythmias). An incremental exercise protocol was used, in which the work rate was increased by 10 W to 20 W min^−1^ after an initial 2 min of unloaded cycle. Standard 12-lead electrocardiograms were obtained continuously. Subjects breathed through a mouthpiece connected to a pneumotachograph. Measurements of mixed expired oxygen, mixed expired carbon dioxide and expired volume were determined at rest and for each breath throughout exercise using a metabolic cart (Jaeger Vyntus CPX). Oxygen uptake (V’O_2_; mL min^−1^; standard temperature and pressure, dry), carbon dioxide production (V’CO_2_; mL/min), gas exchange ratio, minute ventilation (V’E; L/min), respiratory rate, the ventilatory equivalent for carbon dioxide (V’E/V’CO_2_) was determined and averaged every 30 s. Patients were divided in two groups: (1) normal exercise capacity, i.e.VO_2_peak ≥ 80% of predicted values and (2) reduced exercise capacity, i.e. VO_2_peak < 80% of predicted values.

### Pulmonary function testing

Each participant underwent spirometry, plethysmography, and DL_CO_ according to ATS/ERS consensus guidelines [15]. DL_CO_ was measured using the single breath method. K_CO_, which is DL_CO_ corrected for alveolar volume, was used for the analysis. DL_CO_ and K_CO_ were corrected for hemoglobin. The spirometry, lung volumes and DL_CO_ measurements were expressed as percentage of predicted normal values using reference values taken from the prediction equations of the GLI 2012 [16].

### Transthoracic echocardiography at rest

Patients underwent transthoracic echocardiography using Vivid E95 ultrasound system (General Electric). Left ventricular (LV) and left atrial (LA) volumes were measured in two-dimensional apical views according to the biplane Simpson rule. Left ventricular function was assessed using LV ejection fraction (LVEF, biplane Simpson method) and global longitudinal strain (GLS). Mitral inflow velocity pattern, peak velocities of E and A waves and E wave deceleration time were recorded as recommended [17]. Mitral lateral E’ velocities were measured by tissue Doppler imaging. Systolic pulmonary arterial pressure (calculated from tricuspid regurgitation flow) and pulmonary acceleration time (PAcT) was acquired using Doppler method. Right ventricular (RV) size was assessed by RV basal and mid dimensions and diastolic surface and RV function by Tricuspid Annular Plane Systolic Excursion (TAPSE), S’ wave velocity (Doppler tissue imaging), RV ejection delays (RVEDs) and tricuspid regurgitation velocity (TRV) as previously published[18].

### Data presentation and statistical analysis

Continuous variables are reported as mean ± standard deviation (SD) and compared using the unpaired Student t test or the Mann–Whitney test, as appropriate. Qualitative variables are expressed as numbers and percentages and compared with the Chi^2^ or Fischer tests, as appropriate. Pearson correlation coefficients were computed for continuous-continuous variables correlations. Uni- and multivariable linear regression models were used to assess the relationship between VO_2_peak and variables of interests, using a stepwise backward approach for multivariable analysis by first entering all covariates associated with VO_2_peak at the p < 0.2 level in univariate analysis and then removing not significant factors at the p < 0.05 level until the final model was reached. All analyses were performed at the two-tailed P < 0.05 level, using Stata v16.1 (StataCorp, College Station, TX, USA).

## Results

Among the 220 survivors for COVID-19 severe acute pulmonary infection included in COVulnerability cohort, 105 agreed to benefit from a follow-up evaluation three months after hospital discharge. The mean age of patients was 59.2 years and 79 (75.2%) were male. The most common comorbidity was hypertension (41.9%), followed by diabetes (27.6%), and dyslipidemia (20%). Forty-five patients (43.3%) had been admitted to the intensive care unit (ICU) during the acute phase. During hospitalization, 16 patients (15.7%) required high flow nasal cannula (HFNC) and 26 (25.2%) invasive mechanical ventilation (IMV). Other patient’s characteristics and clinical outcomes are shown in Table 1.

**Table 1:** Patient’s characteristics and comparison of clinical factors between patients with a normal and reduced exercise capacity

Parameters	All patients (n = 105)	Normal exercise capacity (n = 68)	Reduced exercise capacity (n = 37)	p-Value
Age, years	59.21 ± 11.81	59.68 ± 12.16	58.35 ± 11.26	0.585
Male (%)	79 (75.2)	46 (67.6)	33 (89.2)	**0.015**
BMI before COVID-19, kg/m^2^	29.11 ± 5.51	30.29 ± 5.64	27.16 ± 4.74	**0.009**
BMI, kg/m^2^	27.92 ± 4.98	29.07 ± 5.24	25.79 ± 3.68	**0.001**
Change in BMI before-after COVID-19, kg/m^2^	− 1.16 ± 1.93	− 0.90 ± 1.59	− 1.59 ± 2.35	0.105
Tobacco history (%)	48 (45.7)	34 (50)	14 (37.8)	0.232
Smoking, pack-years	11.69 ± 16.29	11.5 ± 15.22	12.06 ± 18.38	0.874
mMRC dyspnea scale (0/ 1/ 2/ 3/ 4)	67/ 31/ 5/ 2/ 0	46/ 21/ 1/ 0/ 0	24/ 12/ 1/ 0/ 0	0.61
*Comorbidities*				
Hypertension (%)	44 (41.9)	25 (36.8)	19 (51.4)	0.148
Diabetes (%)	29 (27.6)	12 (17.6)	17 (45.9)	**0.002**
Dyslipidemia (%)	21 (20)	13 (19.1)	8 (21.6)	0.759
Ischemic cardiomyopathy (%)	17 (16.2)	12 (17.6)	5 (13.5)	0.583
Obstructive sleep apnea (%)	15 (14.3)	8 (11.8)	7 (18.9)	0.317
Malignancy (%)	14 (13.5)	8 (11.9)	6 (16.2)	0.541
Chronic kidney disease (%)	11 (10.5)	3 (4.4)	8 (21.6)	**0.006**
COPD (%)	1 (1)	1 (1.5)	0 (0)	0.459
*During COVID-19 hospitalisation*				
ICU stay (%)	45 (43.3)	26 (38.8)	19 (51.4)	0.216
HFNC (%)	16 (15.7)	10 (15.2)	6 (16.7)	0.841
IMV (%)	26 (25.2)	14 (21.2)	12 (32.4)	0.209
Tracheotomy (%)	2 (1.9)	1 (1.5)	1 (2.7)	0.675
ECMO (%)	4 (3.9)	1 (1.5)	3 (8.1)	0.097
Pulmonary embolism (%)	8 (7.8)	7 (10.6)	1 (2.7)	0.150
Acute kidney injury (%)	23 (22.1)	11 (16.2)	12 (33.3)	**0.045**
Cardiogenic shock (%)	2 (1.9)	2 (3)	0 (0)	0.289

Data are presented as n, n (%) or mean ± SD. Bold type represents statistical significance. *BMI* Body mass index, *mMRC* modified Medical Research Council, *COPD* chronic obstructive pulmonary disease, *ICU* intensive care unit, *IMV* invasive mechanical ventilation, *HFNC* high-flow nasal cannula, *ECMO* extracorporeal membrane oxygenation

Despite no major symptoms of dyspnea and 6-MWT in normal range, more than a third of patients (37 patients, 35%) had an impaired CPET with a VO_2_peak under 80%. Interestingly, patients with a reduced exercise capacity did not complain of dyspnea and had similar 6-MWT as compared with those having normal exercise capacity. Compared to patients with normal exercise capacity, patients with reduced exercise capacity were more often men (89.2% vs. 67.6%, p = 0.015), with diabetes (45.9% vs. 17.6%, p = 0.002), renal dysfunction (21.6% vs. 17.6%, p = 0.006) and lower BMI (25.79 ± 3.68 vs. 29.07 ± 5.24, p = 0.001). Of note, smoking habits was similar between groups. The severity of acute COVID-19 disease was not different between groups in terms of ICU admission, HFNC, IMV and ECMO (Table 1). During the exercise test, patients with altered exercise capacity compared to the others reached a lower maximal work, a lower maximal heart rate and had a higher breathing reserve (Table 2). We do not observe desaturation between groups, at the peak of exercise the saturation was 98.4 ± 3.2% in the normal group vs. 99 ± 1.42% in the impaired group (p = 0,239).

**Table 2:** Comparison of pulmonary function, skeletal muscle parameter and transthoracic echocardiography between patients with a normal and reduced exercise capacity

Parameters	All patients (n = 105)	Normal exercise capacity (n = 68)	Reduced exercise capacity (n = 37)	p-Value
*Exercise capacity assessment (CPET)*				
Wrmax, W	116.14 ± 51.67	130.74 ± 53.39	89.32 ± 35.55	**0.0002**
HRmax, bpm	139.43 ± 25.80	143.51 ± 26.38	131.92 ± 23.21	**0.013**
Breathing reserve, %	25.49 ± 19.13	21.14 ± 18.38	33.14 ± 18.23	**0.009**
V'O2max, ml/min	1523.89 ± 552.69	1718.90 ± 540.20	1165.49 ± 368.13	** < 0.0001**
V'CO2max, ml/min	1764.03 ± 696.62	1991.90 ± 700.66	1345.24 ± 458.19	** < 0.0001**
RER	1.15 ± 0.11	1.15 ± 0.09	1.15 ± 0.13	0.979
V'O2max/BW, ml/min/kg	18.28 ± 5.34	20.15 ± 5.16	14.84 ± 3.75	** < 0.0001**
VO_2_max/kg leg muscle mass (ml/kg leg/min)	86.4 ± 21.8	95.7 ± 18.5	68.5 ± 16	** < 0.0001**
Anaerobic threshold %VO2 max predicted	62.7 ± 19.8	71.1 ± 17.4	45.96 ± 12.2	** < 0.0001**
Respiratory frequency, breathe/min	38.98 ± 8.87	39.76 ± 8.56	37.55 ± 9.36	0.319
VO_2_/HR, ml/beat	11.12 ± 3.30	12.18 ± 3.27	9.02 ± 2.17	** < 0.0001**
VO_2_/HR, % predicted	86.32 ± 19.59	96.62 ± 14.69	66.03 ± 9.58	** < 0.0001**
Hemoglobin, g/dl	13.57 ± 1.54	13.99 ± 1.39	12.77 ± 1.52	** < 0.0001**
6-min walking distance, m	485.18 ± 111.08	494.02 ± 109.47	469.22 ± 113.72	0.285
*Pulmonary function*				
FVC, L	3.49 ± 1.11	3.67 ± 1.18	3.17 ± 0.88	**0.026**
FVC, % predicted	84.90 ± 18.08	90.54 ± 16.69	74.54 ± 15.99	** < 0.0001**
FEV1, L	2.78 ± 0.82	2.91 ± 0.83	2.55 ± 0.76	**0.030**
FEV1, % predicted	87.45 ± 18.62	93.26 ± 16.38	76.76 ± 17.92	** < 0.0001**
FEV1/FVC (%)	80 ± 7	81 ± 8	80 ± 7	**0.828**
TLC, L	5.42 ± 1.24	5.57 ± 1.35	5.13 ± 0.93	0.081
TLC, % predicted	83.70 ± 14.72	87.88 ± 13.79	75.81 ± 13.24	** < 0.0001**
DLCO, % predicted	71.27 ± 19.34	74.66 ± 20.48	65.14 ± 15.53	**0.015**
KCO, %	93.87 ± 17.56	94.79 ± 17.59	92.19 ± 17.62	0.472
PaO2, mmHg	89.04 ± 9.35	87.82 ± 9.68	90.81 (8.7)	0.173
PaCO2, mmHg	37.08 ± 4.97	37.21 ± 2.5	36.84 ± 7.24	0.73
*Skeletal muscle mass and function*				
ASMMI, kg/m^2^	7.85 ± 1.17	8.13 ± 1.19	7.36 ± 0.94	**0.001**
Sarcopenia (%)	21 (21)	7 (10.9)	14 (38.9)	**0.001**
Grip test, kg	34.42 ± 10.37	36.33 ± 10.71	31.5 ± 9.27	**0.047**
Pinch test, kg	6.66 ± 2.17	7.04 ± 2.31	6.07 ± 1.82	0.054
*Transthoracic echocardiography*				
CO, L/min	5.71 ± 1.37	5.88 ± 1.44	5.44 ± 1.24	0.182
LVMi, g/m2	84.43 ± 24.5	79.31 ± 18.01	93.43 ± 31.34	**0.012**
LVEF (2D), %	60.25 ± 6.06	60.31 ± 5.75	60.13 ± 6.64	0.898
Global longitudinal strain, %	− 17.24 ± 2.45	− 17.59 ± 2.39	− 16.66 ± 2.49	0.114
E/A ratio	0.89 ± 0.33	0.90 ± 0.34	0.88 ± 0.32	0.767
E/E' ratio	7.1 ± 2.20	7.10 ± 2.03	7.12 ± 2.56	0.971
E' lateral, cm/s	9.46 ± 3.07	9.54 ± 3.36	9.29 ± 2.44	0.733
LAVi, mL	28.15 ± 9.78	27.82 ± 8.77	28.70 ± 11.39	0.697
RVEDs, cm^2^	17.43 ± 4.45	16.70 ± 4.34	18.65 ± 4.45	0.077
TAPSE, mm	21.49 ± 3.33	21.74 ± 3.47	21.04 ± 3.05	0.389
S’ wave, cm/s	12.79 ± 2.08	12.72 ± 2.01	12.95 ± 2.26	0.663
TRV, m/s	2.32 ± 0.34	2.30 ± 0.32	2.35 ± 0.37	0.656
systolic PAP, mmHg	25 ± 6.05	24.61 ± 5.65	25.5 ± 6.66	0.646
PAcT, ms	119.7 ± 27.53	125.84 ± 25.95	106.6 ± 27	**0.024**
RA area, cm^2^	14.68 ± 4.42	14.35 ± 4.42	15.30 ± 4.44	0.387

Data are presented as mean ± SD. Bold type represents statistical significance. *WRmax* maximum work rate, *V'O2max* maximum oxygen uptake, *V'CO2max* maximum carbon dioxide production, *V'O2max/BW* maximum oxygen uptake per kg body weight, *RER* respiratory exchange ratio, *HR* heart rate, *DLCO* diffusing capacity of the lungs for carbon monoxide, *FEV1* forced expiratory volume in the first second, *FVC* forced vital capacity, *KCO* diffusion coefficient, *TCL* total lung capacity, *PaO2* partial pressure of oxygen assessed by blood gas analysis, *PaCO2* partial pressure of carbon dioxide assessed by blood gas analysis, *ASMMI* appendicular skeletal muscle mass index, *PAcT* pulmonary acceleration time, *CO* cardiac output, *LVMi* left ventricular mass index, *LAVi* left atrial volume index, *RVEDs* right ventricular ejection delays, *TAPSE* tricuspid annular plane systolic excursion, *TRV* tricuspid regurgitation velocity, *PAP* pulmonary artery pressure, *RA* right atrium

Reduced exercise capacity was associated with a significant decrease in respiratory function at rest: compared to the normal exercise capacity group, predicted FVC was significantly lower in the impaired group (74.54 ± 15.99% vs. 90.54 ± 16.69%, p < 0.0001) as predicted FEV1 (76.76 ± 17.92% vs. 93.26 ± 16.38%, p < 0.0001) and TLC (75.81 ± 13.24% vs. 87.88 ± 13.79%, p < 0.0001). DL_CO_ was also significantly lower in the reduced exercise capacity group (65.14 ± 15.53% vs. 74.66 ± 20.28%, p = 0.015). K_CO_ was not modified in the reduced exercise capacity group, highlighting a restrictive pulmonary pattern in patients presenting an altered exercise capacity (Table 2).

Furthermore, we found a reduced skeletal muscle mass by ASMMI in the altered exercise capacity group (7.36 ± 0.94 kg/m^2^ vs. 7.85 ± 1.17 kg/m^2^, p = 0.001) together with a decrease in grip test (31.50 ± 9.27 vs. 36.33 ± 10.71, p = 0.047) highlighting an impairment in skeletal muscle strength (Table 2). There also was a decrease in VO_2_/kg of muscle leg in the reduced exercise capacity (68.5 ± 16 vs. 95.7 ± 18.5, p < 0.0001). Notably, sarcopenia was more frequent among low exercise capacity patients compared to the others: 38.9% vs 10.9%, (p = 0.001).

We did not observe any significant cardiac dysfunction with systolic and diastolic parameters in normal range values at rest. Furthermore, LVEF and right ventricular function were similar in both groups (Table 2). Reduced exercise capacity was associated with a shorter PAcT (107 ± 27 ms vs. 126 ± 26 ms, p = 0.02) despite no argument for pulmonary hypertension (based on tricuspid regurgitation peak velocity). Predicted VO_2_/Heart rate was decreased in the altered exercise capacity group (66.03 ± 9.58% vs. 96.62 ± 14.69%, p < 0.0001).

As expected, the univariate analysis showed correlation between VO_2_peak and the predicted values of FEV1, FVC, TLC, DL_CO_ but not with K_CO_ (Table 3, see Additional file 1). Notably, VO_2_peak was also correlated with skeletal muscle mass and grip test. In addition, VO_2_peak was correlated with left ventricle mass index, GLS, E/E’ ratio, RVEDs, TRV and PacT (Table 3, see Additional file 1). Interestingly, in multivariable analysis, age, sex, predicted TLC, ASMMI, GLS and E/E’ ratio, showed an independent correlation with VO2peak (Table 3).

**Table 3:** Multivariable analysis to identify the factors associated with VO_2_peak

	Unadjusted analyses			Multivariable analysis	
Parameters	Correlation coefficient, r	Unadjusted linear regression coefficient (CI95%)	p-Value	Adjusted linear regression coefficient (CI95%)	p-Value
Age, years	0.18	0.004 (0.000;0.008)	0.070	0.01 (0.003–0.012)	**0.001**
Body mass index, kg/m^2^	0.33	0.016 (0.007;0.025)	**0.0006**	(–)	
*Pulmonary function*					
FVC, % predicted	0.52	0.007 (0.005;0.009)	** < 0.0001**	(–)	
FEV1, % predicted	0.51	0.007 (0.005;0.009)	** < 0.0001**	(–)	
TLC, % predicted	0.52	0.009 (0.006;0.012)	** < 0.0001**	0.01 (0.003–0.01)	**0.0004**
DLCO, % predicted	0.38	0.005 (0.003;0.007)	** < 0.0001**	(–)	
*Skeletal muscle mass and function*					
ASMMI, kg/m^2^	0.34	0.072 (0.032;0.113)	**0.0006**	0.09 (0.05–0.12)	** < 0.0001**
Grip test, kg	0.25	0.006 (0.001;0.011)	**0.027**	(–)	
*Transthoracic echocardiography*					
LVMi, g/m^2^	− 0.29	− 0.003 (− 0.005; − 0.001)	**0.009**	(–)	
PAcT, ms	0.35	0.003 (0.001;0.005)	**0.017**	(–)	

Bold type represents statistical significance. *DLCO* diffusing capacity of the lungs for carbon monoxide, *FEV1* forced expiratory volume in the first second, *FVC* forced vital capacity, *KCO* diffusion coefficient, *TCL* total lung capacity, *PaO2* partial pressure of oxygen assessed by blood gas analysis, *PaCO2* partial pressure of carbon dioxide assessed by blood gas analysis, *ASMMI* appendicular skeletal muscle mass index, *PAcT* pulmonary acceleration time, *CO* cardiac output, *LVMi* left ventricular mass index, *LAVi* left atrial volume index, *RVEDs* right ventricular ejection delays, *TAPSE* tricuspid annular plane systolic excursion, *TRV* tricuspid regurgitation velocity, *PAP* pulmonary artery pressure, *RA* right atrium

Finally, we identified a slight but statistically significant increase in circulatory inflammatory biomarkers (CRP, IL6, TNFα) in patients with reduced VO_2_peak compared to normal exercise capacity group (Table 4). There was a negative correlation between VO2peak and IL6 (r = − 0.24, p = 0.013), TNFa (r = − 0.34, p = 0.0003) and CRP (r = − 0.29, p = 0.003).

**Table 4:** Comparison of circulatory inflammatory biomarkers between patients with normal and reduced exercise capacity

Parameters (normal values)	All patients (n = 105)	Normal exercise capacity (n = 68)	Reduced exercise capacity (n = 37)	p-Value
CRP, mg/L (< 5 mg/l)	3.61 ± 6.00	2.46 ± 4.26	5.79 ± 7.99	**0.012**
Interleukin-6, pg/ml (< 7 pg/l)	5.80 ± 7.58	5.17 ± 7.21	6.96 ± 8.18	**0.013**
Interleukin-8, pg/ml (6.7–16.2 pg/ml)	116.03 ± 249.6	131.24 ± 299.17	88.06 ± 110.27	0.835
TNFα pg/ml (4.05–8.34 pg/ml)	19.36 ± 8.87	17.3 ± 7.82	23.14 ± 9.51	** < 0.0001**

Data are presented as mean ± SD. Bold type represents statistical significance. *CRP* C reactive protein, *TNFα* tumor necrosis factor α

## Discussion

Three months after hospitalization for a severe COVID-19 pulmonary infection, we show that 35% of survivors of COVulnerability cohort have a clinically occult impaired exercise capacity, which was unmasked by CPET whereas clinical symptoms and 6-MWT were not contributive. Beyond alteration of pulmonary function, exercise limitation was associated to sarcopenia and reduced skeletal muscle function but no significant cardiac dysfunction.

Our results highlight the importance to perform a global and systematic evaluation of severe patients during follow up, including CPET to detect exercise limitation beyond the existence of lung sequelae. Indeed, the initial disease severity during the acute hospitalization was not related to the exercise capacity at 3 months, highlighting the complexity to predict which patient will develop an exercise limitation. Furthermore, CPET is able to unmask an exercise limitation that cannot be identified by dyspnea measure or 6-MWT [19]. In line with this observation, we showed that these two latter criteria were similar between normal and impaired exercise capacity groups and were not contributive for the diagnosis of exercise limitation. Thus, our study adds a piece of evidence for the high rate of exercise limitation in COVID-19 survivors as previously reported in coronavirus outbreak [4, 20, 21] and more recently identified in COVID-19 patients [10, 11, 22].

Once raising awareness of exercise limitation in the follow-up of COVID-19 patients, we aimed to decipher the underlying mechanisms by performing a comprehensive multi-organ evaluation to identify factors associated with a reduced exercise capacity. Whilst most of studies are focusing on lung alterations [11, 22], our study is the first to identify sarcopenia as a main contributor. Indeed, a reduced exercise capacity was associated with a decrease in muscle mass and function assessed by ASMMI and grip test. The reduction of VO2/kg leg muscle mass in the group with a reduced exercise capacity suggest strongly that the muscle is dysfunctional. The reduction of anaerobic threshold is also an argument for an alteration of muscle function responsible of the exercise limitation. Moreover, sarcopenia was very common among patients with low exercise capacity, thus clarifying the suspicion of muscular deconditioning reported by previous studies [11]. Such a link between exercise limitation and sarcopenia has been reported in chronic cardiac and lung diseases [23, 24]. However, in our cohort, COVID-19 survivors with an exercise limitation had a higher rate of sarcopenia (almost 40%) compared with these diseases (15–34%) [25–27] highlighting the important chronic impact of COVID-19 on muscle mass. Based on these results, prevention and treatment of sarcopenia must be considered during follow up of COVID-19 survivors.

If obesity and high BMI are clearly associated with higher frequency of severe COVID-19 infections and worse prognosis in the acute phase [28, 29], we showed that patients with low BMI may be more vulnerable in the follow-up phase with a lower exercise capacity.

We also found that an impaired exercise capacity was associated with a lower lung function and particularly with a decrease in lung volume that characterized COVID-19 survivors [8, 9, 22, 30, 31]. A reduction of gas transfer measured by DL_CO_ was also present, but this difference disappeared when this value was related with alveolar ventilation (K_CO_), confirming the restrictive pulmonary pattern in patients presenting a decreased effort capacity. However, the breathing reserve at VO_2_peak was higher in the reduced exercise group suggesting that this restrictive profile is not responsible of the exercise limitation.

Echocardiography at rest suggest that the role of cardiac dysfunction can be ruled out regarding the lack of systolic and diastolic function abnormalities and the weak correlation between systolic and diastolic parameters with VO_2_peak. However, we cannot exclude a cardiac dysfunction during exercise based on VO2/heart rate in patients with impaired exercise capacity. Another explanation to the VO2/heart reduction could be the reduction of peripheral extraction associated with muscle dysfunction. Further explorations such effort cardiac echography would be interested to determine the impact of cardiac dysfunction, but it was not in the scope of this study.

We also observed that inflammatory circulating biomarkers are higher in patients with low exercise capacity. That could be an important component of the phenotype of patients with low exercise capacity associated with altered lung function and muscle alteration. Lower muscle mass may be related to persistent systemic inflammation from acute COVID-19 infection [32, 33] to chronic sequelae [30]. Indeed, a high level of circulating inflammatory biomarkers, related to a chronic inflammation, is known to be associated with lower skeletal muscle strength and muscle mass [34, 35] and can participate to the deconditioning process observed in our patients. The higher level of IL-6 and TNFα in the reduced exercise capacity group at 3 months of the acute disease highlight a persistent inflammatory signature. These results are consistent with the IL-6 and TNFα serum levels that are described to be independent and significant predictors of COVID-19 disease severity [36]. Decrease in hemoglobin level in the reduced exercise capacity group can be linked to this persistent inflammation.

One limitation of our study includes the absence of preexisting evaluation of these patients before the acute phase of the disease and organ alteration that may precede the hospitalization. For example, sarcopenia could be induced by diabetes prior to COVID-19 infection. BMI values before COVID-19 infection showed a difference between groups, it can limit the interpretation and allow to hypothesize that other parameters, like a preexisting muscle mass and function alteration, was already present between groups.

## Conclusion

At 3 months of a severe COVID-19 pulmonary infection, one third of patients enrolled in COVulnerability cohort have an exercise limitation. This limitation is associated with a lung and muscular dysfunction. Adapted rehabilitation for these patients could decrease the global sequelae of this disease.

## Supplementary Information

**Additional file 1: Table S1.** Univariate and multivariable analysis to identify the factors associated with VO2peak.

## Data Availability

The datasets used and/or analysed the current study are available from the corresponding author on reasonable request.

## Abbreviations

6-MWT: 6-Minute walk test
ASMMI: Appendicular skeletal muscle mass index
BMI: Body-mass index
CO: Cardiac output
COVID-19: Coronavirus disease 2019
CPET: Cardiopulmonary exercise testing
DL_CO_: Diffusing capacity of the lungs for carbon monoxide
ECMO: Extracorporeal membrane oxygenation
FEV1: Forced expiratory volume in the first second
FVC: Forced vital capacity
GLS: Global longitudinal strain
HFNC: High-flow nasal cannula
HRmax: Maximum heart rate
ICU: Intensive care unit
IMV: Invasive mechanical ventilation
K_CO_: Diffusion coefficient
LAVi: Left atrial volume index
LVEF: Left ventricle ejection fraction
LVMi: Left ventricular mass index
PaO_2_: Partial pressure of oxygen assessed by blood gas analysis
PaCO_2_: Partial pressure of carbon dioxide assessed by blood gas analysis
PAcT: Pulmonary acceleration time
PAP: Pulmonary artery pressure
RA: Right atrium
RER: Respiratory exchange ratio
RV: Right ventricle
TAPSE: Tricuspid Annular Plane Systolic Excursion
TCL: Total lung capacity
TNFα: Tumor necrosis factor α
TRV: Tricuspid regurgitation velocity
V'CO2max: Maximum carbon dioxide production
V'O2max: Maximum oxygen uptake
V'O2max/BW: Maximum oxygen uptake per kg body weight
WRmax: Maximum work rate
