# Supplementary material for: Identification of factors impairing exercise capacity after severe COVID-19 pulmonary infection: a 3-month follow-up of prospective COVulnerability cohort
Source: Respir Res. 2022 Mar 22;23:68. doi: 10.1186/s12931-022-01977-z (PMC8938727; doi:10.1186/s12931-022-01977-z)
Supplement: Supplementary file 1 — Additional file 1: Table S1. Univariate and multivariable analysis to identify the factors associated with VO2peak. [file 12931_2022_1977_MOESM1_ESM.docx]

Additional File 1:

Table S1: Univariate and multivariable analysis to identify the factors associated with VO2peak.

|  |  | **Unadjusted analyses** | | |  | **Multivariable analysis** | |
| --- | --- | --- | --- | --- | --- | --- | --- |
| Parameters |  | Correlation coefficient, r | Unadjusted linear regression coefficient (CI95%) | p-Value |  | Adjusted linear regression coefficient (CI95%) | p-Value |
| Age, years |  | 0.18 | 0.004 (0.000;0.008) | 0.070 |  | 0.01 (0.003 - 0.012) | **0.001** |
| Body mass index, kg/m2 |  | 0.33 | 0.016 (0.007;0.025) | **0.0006** |  | (-) |  |
| Smoking, pack-years |  | -0.07 | -0.001 (-0.004;0.002) | 0.509 |  | (-) |  |
|  |  |  |  |  |  |  |  |
| **Pulmonary function** |  |  |  |  |  |  |  |
| FVC, % predicted |  | 0.52 | 0.007 (0.005;0.009) | **<0.0001** |  | (-) |  |
| FEV1, % predicted |  | 0.51 | 0.007 (0.005;0.009) | **<0.0001** |  | (-) |  |
| TLC, % predicted |  | 0.52 | 0.009 (0.006;0.012) | **<0.0001** |  | 0.01 (0.003 - 0.01) | **0.0004** |
| DLCO, % predicted |  | 0.38 | 0.005 (0.003;0.007) | **<0.0001** |  | (-) |  |
| KCO, % |  | 0.05 | 0.001 (-0.002;0.003) | 0.604 |  | 0.002 (-0.0007 - 0.004) | 0.145 |
| PaO2, mmHg |  | -0.03 | -0.001 (-0.007;0.006) | 0.813 |  | (-) |  |
| PaCO2, mmHg |  | 0.00 | 0.000 (-0.012;0.012) | 0.969 |  | (-) |  |
|  |  |  |  |  |  |  |  |
| **Skeletal muscle mass and function** | | |  |  |  | (-) |  |
| ASSMI, kg/m2 |  | 0.34 | 0.072 (0.032;0.113) | **0.0006** |  | 0.09 (0.05 - 0.12) | **<0.0001** |
| Grip test, kg |  | 0.25 | 0.006 (0.001;0.011) | **0.027** |  | (-) |  |
| Pinch test, kg |  | 0.20 | 0.022 (-0.003;0.047) | 0.090 |  | (-) |  |
|  |  |  |  |  |  |  |  |
| **Transthoracic echocardiography** | |  |  |  |  |  |  |
| CO, L/min |  | 0.06 | 0.011 (-0.031;0.052) | 0.611 |  | (-) |  |
| LVMi, g/m2 |  | -0.29 | -0.003 (-0.005;-0.001) | **0.009** |  | (-) |  |
| LVEF (2D), % |  | 0.12 | 0.005 (-0.004;0.014) | 0.288 |  | (-) |  |
| Global longitudinal strain, % |  | -0.24 | -0.023 (-0.045;-0.001) | **0.038** |  | -0.02 (-0.04 - -0.01) | **0.004** |
| E/A ratio |  | -0.13 | -0.090 (-0.251;0.071) | 0.271 |  | (-) |  |
| E/E' ratio |  | -0.24 | -0.026 (-0.051;-0.002) | **0.036** |  | -0.03 (-0.05 - -0.01) | **0.008** |
| E' lateral, cm/s |  | 0.09 | 0.007 (-0.011;0.025) | 0.427 |  | (-) |  |
| LAVi, mL |  | -0.18 | -0.004 (-0.010;0.001) | 0.114 |  | (-) |  |
| RVEDs, cm² |  | -0.28 | -0.015 (-0.027;-0.003) | **0.018** |  | (-) |  |
| TAPSE, mm |  | 0.07 | 0.005 (-0.012;0.021) | 0.555 |  | (-) |  |
| S’ wave, cm/s |  | -0.04 | -0.004 (-0.033;0.024) | 0.757 |  | (-) |  |
| TRV, m/s |  | -0.37 | -0.256 (-0.450;-0.063) | **0.010** |  | (-) |  |
| systolic PAP, mmHg |  | -0,30 | -0.012 (-0.025;0.000) | 0.059 |  | (-) |  |
| PAcT, ms |  | 0.35 | 0.003 (0.001;0.005) | **0.017** |  | (-) |  |
| RA area, cm² |  | -0.01 | -0.005 (-0.018;0.007) | 0.402 |  | (-) |  |

Bold type represents statistical significance. WRmax: maximum work rate; V'O2max: maximum oxygen uptake; V'CO2max: maximum carbon dioxide production; V'O2max/BW: maximum oxygen uptake per kg body weight; RER: respiratory exchange ratio; HRmax: maximum heart rate; DLCO: diffusing capacity of the lungs for carbon monoxide; FEV1: Forced expiratory volume in the first second; FVC: forced vital capacity; KCO: diffusion coefficient; TCL: total lung capacity; PaO2: partial pressure of oxygen assessed by blood gas analysis; PaCO2: partial pressure of carbon dioxide assessed by blood gas analysis; ASSMI: Appendicular skeletal muscle mass index; PAcT: Pulmonary acceleration time, CO: cardiac output, LVMi: left ventricular mass index, LAVi: left atrial volume index , RVEDs: Right ventricular ejection delays, TAPSE: Tricuspid Annular Plane Systolic Excursion, TRV: tricuspid regurgitation velocity, PAP: pulmonary artery pressure , RA: right atrium
